# Relationship between G1287A of the NET Gene Polymorphisms and Brain Volume in Major Depressive Disorder: A Voxel-Based MRI Study

**DOI:** 10.1371/journal.pone.0150712

**Published:** 2016-03-09

**Authors:** Issei Ueda, Shingo Kakeda, Keita Watanabe, Reiji Yoshimura, Taro Kishi, Osamu Abe, Satoru Ide, Junji Moriya, Asuka Katsuki, Hikaru Hori, Nakao Iwata, Jun Nakamura, Yukunori Korogi

**Affiliations:** 1 Department of Radiology, University of Occupational and Environmental Health, Kitakyushu, Japan; 2 Department of Psychiatry, University of Occupational and Environmental Health, Kitakyushu, Japan; 3 Department of Psychiatry, Fujita Health University, School of Medicine, Toyoake, Japan; 4 Department of Radiology, Nihon University School of Medicine, Tokyo, Japan; Benito Menni Complejo Asistencial en Salud Mental, SPAIN

## Abstract

**Background:**

Earlier studies implicated norepinephrine transporter (NET) gene (*SLC6A2*) polymorphisms in the etiology of major depressive disorder (MDD). Recently, two single nucleotide *SLC6A2* polymorphisms, G1287A in exon 9 and T-182C in the promoter region, were found to be associated with MDD in different populations. We investigated the relationship between the brain volume and these two polymorphisms of the *SLC6A2* in MDD patients.

**Methods:**

We obtained 3D high-resolution T1-weighted images of 30 first-episode MDD patients and 48 age- and sex-matched healthy subjects (HS). All were divided into 4 groups based on polymorphism of either the G1287A or the T-182C genotype. VBM analysis examined the effects of diagnosis, genotype, and genotype-diagnosis interactions.

**Results:**

Diagnosis effects on the brain morphology were found in the left superior temporal cortex. No significant genotype effects were found in the T-182C and the G1287A. A significant genotype (G1287A)–diagnosis interaction was found in the left dorsolateral prefrontal cortex. No significant genotype (T-182C)–diagnosis interaction effects were observed in any brain region.

**Conclusions:**

In MDD patients there seems to be a relationship between the volume of the dorsolateral prefrontal cortex and polymorphism of the *SLC6A2* G1287A gene.

## Introduction

Norepinephrine (NE) is a monoamine neurotransmitter implicated in various behavioral and psychological functions including learning and memory, anxiety, arousal, and mood, as well as other disorders such as addiction, depression, and attention deficit/hyperactivity disorder [1, 2]. The norepinephrine transporter (NET), which is also known as solute carrier family 6 member 2 (SLC6A2), is responsible for norepinephrine re-uptake by the presynaptic terminal, and is a target for tricyclic antidepressants, selective norepinephrine re-uptake inhibitors, and serotonin-NE re-uptake inhibitors used to treat major depressive disorder (MDD) [3, 4]. It is also been suggested that the NET is involved in the pathogenesis of MDD itself [5].

The NET gene (*SLC6A2*, OMIM *163970; 19 exons in this genomic region spanning 50.589 kb) is located on chromosome 16q12.2. Among the several known *SLC6A2* polymorphisms, most studies on the etiology of MDD have focused on T-182C (rs2242446) in the 5’-flanking promoter region and G1287A (rs5569) in exon 9. Because the promoter region of *SLC6A2* contains several cis-elements that play a critical role in transcription regulation [6, 7], changes in the DNA structure of this promoter may lead to altered transcriptional activity. Jonsson and colleagues reported that healthy subjects (HS) with the G/G genotype of the G1287A had higher cerebrospinal fluid concentration of the NE metabolite 3-methoxy-4-hydroxyphenylglycol (MPHG) compared to other genotypes [8]. Recently, these single nucleotide polymorphisms (SNPs) were found to be associated with MDD [9, 10]. Studies on the relationship between susceptibility to MDD and *SLC6A2* polymorphisms suggested that they may confer differential sensitivity to specific antidepressant treatments [11, 12]. Indeed, different combinations of *SLC6A2* polymorphisms may be associated with distinct sub-phenotypes of MDD; there was a dose relationship between the number of T containing genotypes and the presence of recurrent depression [13].

Morphological brain abnormalities in MDD patients may be attributable to genetic- and epigenetic factors that regulate brain development and neurodegeneration. For instance, some studies in MDD patients have yielded evidence of a relationship between brain volume and genetic factors, including particularly brain-derived neurotrophic factor (BDNF) and methylenetetrahydrofolate reductase (MTHFR)/catechol-O-methyltransferase (COMT) polymorphisms[14–18]. However, to the best of our knowledge, no previous studies have examined neuroimaging changes associated with SLC6A2 polymorphisms in MDD patients. We investigated the relationship between the brain volume and T-182C and G1287A of the *SLC6A2* in MDD.

## Materials and Methods

### Study Participants

The protocol of this prospective study was approved by the Ethics Committee of the University of Occupational and Environmental Health. All participants provided prior written informed consent for participation in this study. We recruited 30 Japanese, right-handed, treatment-naive first-episode patients with MDD from the in-patient and out-patient services of the University Hospital of Occupational and Environmental Health. A psychiatrist (K.H.) with 7 years’ experience diagnosed the patients using the Structured Clinical Interview for DSM-IV (SCID).The severity of depression was evaluated using the 17-item Hamilton Rating Scale for Depression (HAMD17). Only patients with a HAMD17 score ≥ 14 were eligible for the study. Exclusion criteria included any history of neurological or other physical diseases and comorbidities with other disorders (i.e., there should be no evidence of schizoaffective disorder, bipolar disorder, Axis II, personality disorders, or mental retardation). We also recruited 48 Japanese HS from nearby communities, who included staff at our institution and also their relatives by blood or marriage and friends. They were interviewed by the same psychiatrist using the SCID for DSM-IV, non-patient edition [19].

The 30 MDD patients and the 48 HS were divided into groups based on their G1287A and their T-182C genotype. With respect to G1287A, there were 12 MDD patients with the G/G- and 18 with the A/- genotype (G/A n = 13, A/A n = 5); 27 HS had the G/G and 21 the A/- genotype (G/A n = 17, or A/A n = 4) (Table 1). The 78 participants were also divided into groups based on their T-182C genotype; 11 MDD patients had the T/T genotype and 19 the C/- genotype (T/C n = 10, C/C n = 9). Of the 48 HS, 27 read the T/T and 21 the C/- genotype (T/C n = 17, C/C n = 4) (Table 1).

**Table 1:** Demographic and Clinical Characteristics of Participants.

			Age	Female number	Years of education*	Total gray matter volume [ml]*	HAMD total score
			mean (sd)		mean (sd)	mean (sd)	mean (sd)
ALL	HS		41.2 (11.4)	13	16.6 (3.0)	698.9 (61.4)	- (-)
	(n = 48)						
	MDD		44.3 (13.0)	13	13.4 (2.5)	669.2 (64.9)	7.7 (5.1)
	(n = 30)						
G1287A	HS	G/G (n = 27)	40.3 (9.1)	6	16.7 (2.9)	711.6 (66.3)	- (-)
	(n = 48)						
		A/- (n = 21)	42.2 (14.0)	7	16.3 (3.1)	682.5 (51.5)	- (-)
		G/A = 17, A/A = 4]					
	MDD	G/G (n = 12)	41.0 (11.7)	4	12.8 (1.8)	644.4 (41.8)	7.8 (4.0)
	(n = 30)						
		A/- (n = 18)	47.6 (13.4)	9	13.8 (2.9)	656.6 (72.8)	7.6 (6.0)
		[G/A = 13, A/A = 5]					
T-182C	HS	T/T (n = 27)	42.8 (10.3)	4	16.5 (2.2)	713.2 (40.7)	- (-)
	(n = 48)						
		C/- (n = 21)	40.3 (12.7)	9	16.5 (3.8)	680.4 (66.9)	- (-)
		[T/C = 17, C/C = 4]					
	MDD	T/T (n = 11)	46.3 (13.9)	5	13.2 (3.0)	661.1 (73.1)	5.5 (3.4)
	(n = 30)						
		C/- (n = 19)	44.2 (12.7)	8	13.5 (2.3)	673.8 (61.2)	8.9 (5.6)
		[T/C = 10, C/C = 9]					

Abbreviations: sd = standard deviation; HS = healthy subjects; MDD = Major depression disorders; HAMD = 17-item Hamilton Rating Scale for Depression; G = Guanine; A = Adenine; T = Thymine; C = Cytosine.

*There was a significant difference between HS and MDD (p < 0.01).

### Genotyping

All 78 participants underwent neuroimaging; they also provided a blood sample from which DNA was extracted according to standard laboratory protocols. DNA was isolated from peripheral blood mononuclear cells using the QIAamp DNA Mini-Kit (QIAGEN, Tokyo, Japan). Genotyping was carried out with the polymerase chain reaction (PCR) SNP genotyping system using the BigDye Terminator v3.1 Cycle Sequencing Kit (Life Technologies Japan, Tokyo, Japan. The DNA was read using a BMG Applied Biosystem 3730xI DNA Analyzer (Applied Bioststem, Forest City, CA, USA), T-182C in the promoter region was determined with a modification of the method of Zill et al. [9] and G1287A in exon 9 with the method of Jönsson et al. [8]. In brief, the cycling conditions were an initial denaturation at 94°C for 4minutes, followed by 35 cycles of 94l denatu5 seconds, 59°9econds5 seconds, 72lowed by5 seconds, and a final elongation at 72denatur4 minutes. PCR reactions for SNP rs5569 were performed in a total volume of 20 μL, containing 20 ng of genomic DNA, 200 μM dNTPs, 0.2 μM each primer, 2.5 μL 10 volume of 2fe Takara Bio, Tokyo, Japan), and 1 unit of Taq DNA polymerase (Takara Bio, Tokyu, Japan). Forward primer (T-182A): 5-CTCCTGTGGCTGTTGAAGTGT-3; reverse primer (T-182A): 5 –GCTGGCGAGAGGAACTTTAC-3; forward primer (G1287A): 5-GACAGGTAGCTGTTGCGTAGG; reverse primer (G1287)A: 5-CCCAGCCTCTACCTGG-3.

### MRI and Image Processing for Voxel-Based Morphometry (VBM)

Magnetic resonance imaging (MRI) data were obtained on a 3T scanner (Signa EXCITE 3T; GE Healthcare, Milwaukee, WI, USA) using a dedicated eight-channel phased-array coil (USA Instruments Aurora, OH, USA). For three-dimensional fast-spoiled gradient-recalled acquisition with steady state (3D-FSPGR) the parameters were: repetition time msec/echo time msec/inversion time, 10/4.1/700; flip angle, 10°; field of view 24 cm; section thickness, 1.2 mm; resolution, 0.9 x 0.9 x 1.2 mm. All images were corrected for image distortion due to gradient non-linearity using “GradWarp” [20] and for intensity inhomogeneity using “N3” [21]. Image processing for VBM [22, 23], a fully automatic technique used for the computational analysis of differences in regional brain volumes throughout the entire brain, was with SPM8 (Statistical Parametric Mapping 8; Institute of Neurology, London, UK). The 3D-FSPGR images in native space were spatially normalized and segmented into gray matter (GM), white matter, and cerebrospinal fluid (CSF) images, and intensity-modulated using the DARTEL (Diffeomorphic Anatomical Registration Through Exponential Lie Algebra) toolbox in a high-dimensional normalization protocol. The DARTEL toolbox has been proposed by Ashburner [24] as an alternative method for normalization in SPM. In an intensity-modulation step, the voxel values of the segmented images were multiplied by the measure of the warped and unwarped structures derived from the nonlinear step of the spatial normalization. This step converted the relative regional GM density into the absolute GM density expressed as the amount of GM per unit volume of brain tissue before spatial normalization. The resulting modulated gray and white matter images were smoothed with an 8-mm Gaussian kernel.

### Statistical Analysis

Demographic and clinical characteristics of the two groups were compared using t-tests (unpaired, two tailed) and chi-squared as appropriate. Total GM volume was also compared between the groups using an unpaired two tailed t-test.

Genotype deviation from the Hardy-Weinberg equilibrium (HWE) was evaluated by chi-square test (SAS/Genetics, release 8.2, SAS Japan Inc, Tokyo, Japan).

For VBM analysis, statistical analyses were performed using the SPM8 software program. Morphological changes in the GM were assessed using a full factorial model with the diagnosis and genotype status (G1287A: G/G or A/-; T-182C: T/T or C/-) set as independent variables. Age, sex, total GM volume and years of education were included as covariates of no interest. The comparisons made within the 2 x 2 factorial design were:

1. Diagnosis effects, MDD vs HS,
2. Genotype effects, G1287A: G/G genotype participants (MDD and HS) vs A/- genotype participants, T-182C: T/T genotype participants vs C/- genotype participants,
3. Genotype—diagnosis interaction, G1287A: diagnosis effects in G/G genotype participants vs diagnosis effects in A/- genotype participants, T-182C: diagnosis effects in T/T genotype participants vs diagnosis effects in C/- genotype participants.

The cluster-level threshold was set at familywise error (FWE)-corrected P < 0.05, with a voxel-level threshold set at uncorrected-P < 0.001.

## Results

### Demographic and Clinical Data

Genotype frequencies were in Hardy Weinberg equilibrium (G1287A of HS: df = 1, χ2 = 0.3115, p > 0.05; G1287A of MDD: df = 1, χ2 = 0.209, p > 0.05; T-182C of HS: df = 1, χ2 = 0.3115, p > 0.05; T-182C of MDD: df = 1, χ2 = 3.274, p > 0.05). While there were no significant differences with regard to the distribution of age and sex between the HS and the MDD patients, there were significant differences in the total GM volume and the years of education (p < 0.01) between the groups. (Table 1)

### VBM analysis

#### (a) Diagnosis effects: HS vs MDD

**Fig 1:**
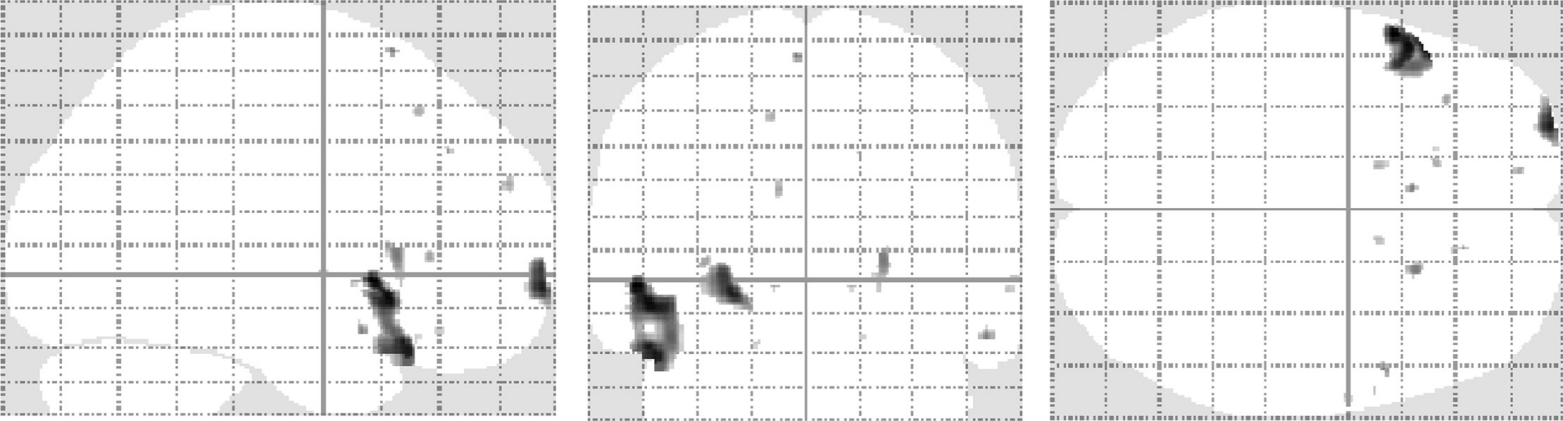
Results of the analysis of the diagnosis effects (HS versus MDD). The images show statistical parametric maps {SPM (t)}. The volume of the superior temporal cortex is significantly smaller in the MDD patients than in the HS (FWE corrected p = 0.02).

The volume of the left superior temporal cortex was significantly smaller in MDD patients than the HS (FWE corrected p = 0.02, T value = 4.28, MNI = [–55, 13, –4]) (Fig 1, Table 1).

#### (b) Genotype effects

In the G1287A, no brain regions showed any significant differences in the GM volume between G/G and A/- genotype participants. In the T-182C, no brain regions also showed significant differences in the GM volume between T/T and C/- genotype participants.

#### (c) Genotype—diagnosis interaction

**Fig 2:**
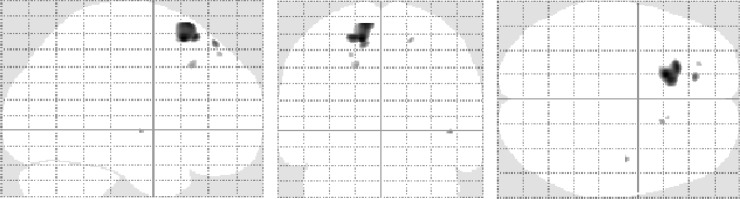
Statistical parametric maps of the analysis of the G1287A genotype—diagnosis interaction. The images show statistical parametric maps {SPM (t)}. Significant volume reduction in the left dorsolateral prefrontal cortex associated with MDD was observed in the G/G genotype participants compared with the A/- genotype participants (FWE corrected p = 0.03).

**Fig 3:**
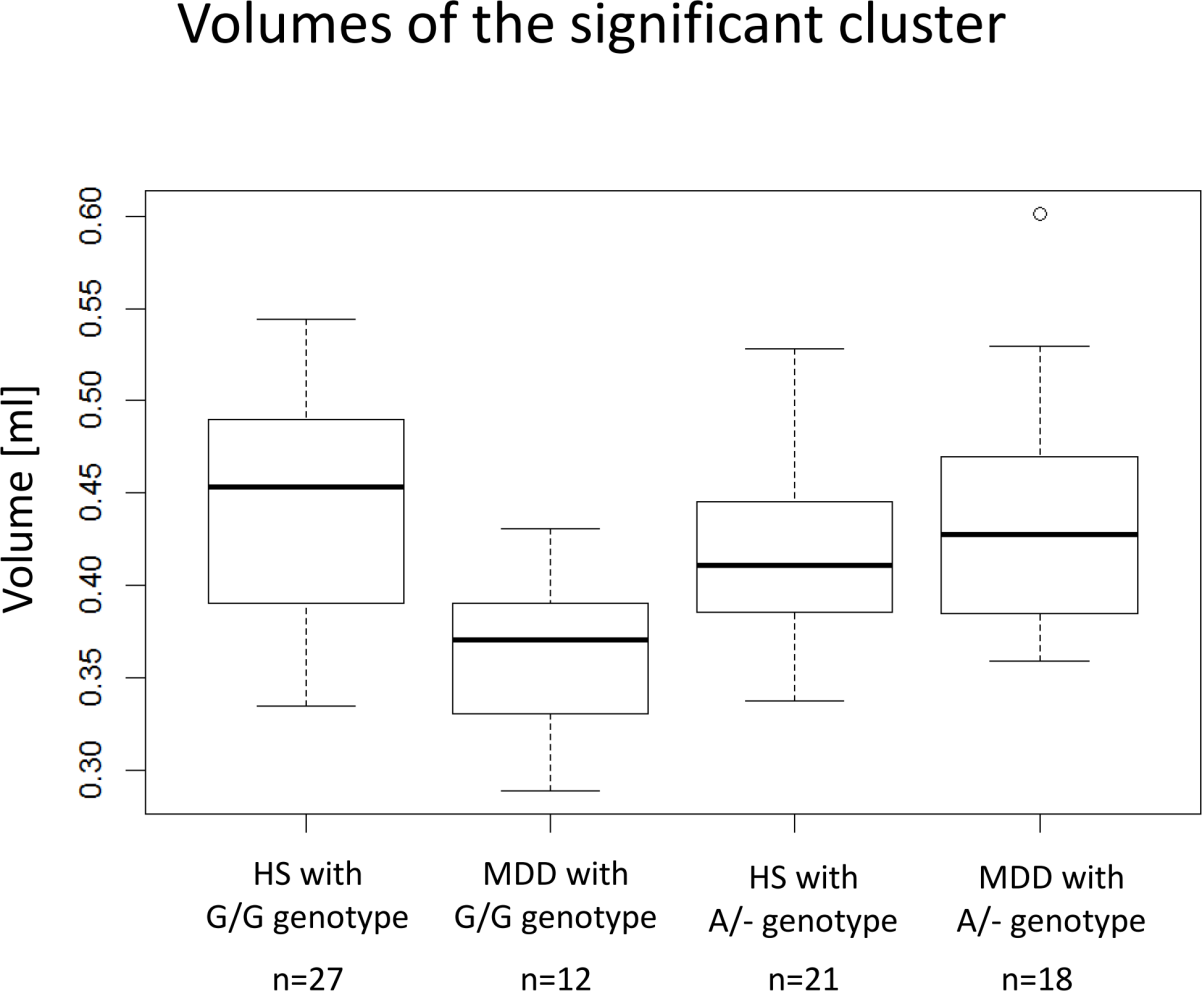
Box plots of the analysis of the G1287A genotype—diagnosis interaction. These box plots show the medians, quartiles and ranges of the GM volumes of the cluster in left dorsolateral prefrontal cortex. The dot at the "MDD with A/- genotype" represent an outlier.

For G1287A, we found a significant genotype—diagnosis interaction in relation to brain morphology. In comparison with A/- genotype participants, G/G genotype participants demonstrated significantly larger volume in the left dorsolateral prefrontal cortex (PFC); in other words, the significant volume change in the left dorsolateral PFC associated with MDD was observed in the G/G genotype participants compared with the A/- genotype participants (FWE corrected p = 0.03, T value = 4.37, MNI = [–15, 18, 56]) (Figs 2 and 3 and Table 2).

**Table 2:** Results of VBM analysis.

Anatomical regions	FDR corrected p	uncorrected p	Cluster size	T-value	Talairach coordinates		
	(culuster level)	(culuster level)		(Voxel level)	x	y	z
**Diagnosis effects (MDD < HS)**							
Left temporal pole	0.021	0.007	1737	4.28	-50	23	-23
				4.19	-47	16	-10
				4.1	-56	17	-24
**Genotype (*SLC6A2* G1287A)-diagnosis interaction**							
Left superior frontal gyrus	0.031	0.01	1523	4.37	-15	18	56
				4.18	-13	20	66
				4.16	-19	26	57

Abbreviations: MDD = Major depression disorders; HS = healthy subjects; NET = Norepinephrine transporter.

For T-182C, there were no significant genotype—diagnosis interactions in relation to brain morphology.

## Discussion

This study provides, to our knowledge, the first evidence of a relationship between brain volume and polymorphsisms in the NET gene in MDD patients. Specifically, our analysis of the genotype—diagnosis interaction for G1287A was significant, with volume in the left dorsolateral PFC being larger in MDD patients with the G/G genotype than in HS participants with this genotype.

While we did not find that the G1287A polymorphism influenced brain structure in the patients or the controls (ie there was no main effect of genotype), we did find a significant genotype x diagnosis interaction. In current study, we found that, for the G1287A, no brain regions showed any significant differences in the GM volume between G/G- and A/- genotype participants. In the diagnosis effects, as suggested in the previous VBM [25, 26] and functional MRI [27] studies, MDD patients showed significant smaller volume in left superior temporal cortex which plays a crucial role in emotional processing [28]. However, the detected brain region (left superior temporal cortex) by simple comparison between the MDD and the HS was different from the detected brain region (PFC) by the genotype-diagnosis interaction. These results may also support the conclusion that the G1287A was associated with volume change of the PFC in the MDD patients.

In MDD the PFC, in particular the dorsolateral PFC, has been a focus of imaging studies. Therefore, our findings are in line with many neuroimaging studies that demonstrated the presence of abnormalities in the PFC in MDD patients; Koolschijn et al. [29] and Bora et al. [30] conducted meta-analysis of VBM studies for gray matter abnormalities in MDD and demonstrated smaller PFC volume in MDD patients, although other areas such as the hippocampus, putamen, and caudate have also been found in these meta-analyses. Moreover, Drevets et al showed decreased cerebral blood flow and glucose metabolism in the orbitofrontal and medial prefrontal cortex of the PFC by positron emission tomography (PET) [31]. According to the authors of the PET study, the decrease in activity is explained by the corresponding volume reduction of the PFC. Further, although it is controversial [32], previous studies with stroke patients suggest that depression results from left frontal lobe lesions [33–35].

Other studies reported an association between the NET in the PFC and the drug effects to psychiatric disorders [36–38], although the underlying mechanisms of action are not fully understood. The effects of methylphenidate, a first-line psychostimulant medication for attention-deficit/hyperactivity disorder (ADHD) [36], affects extracellular dopamine and norepinephrine dynamics in the PFC by inhibiting NET. In ADHD patients, methylphenidate improved PFC-dependent behavioral and cognitive processes including behavioral inhibition, working memory, and planning [37]. In patients with MDD the antidepressant drug reboxetine selectively blocked NET and improved their information memory with a positive valence [38]. Interestingly, animal studies revealed that reboxetine increased the level of dopamine, the precursor of norepinephrine, in the PFC [39, 40]. Therefore, our results also support the level of dopamine in the PFC as one of the elements involved in the pathophysiology of psychiatric disorders including MDD.

In our study the G1287A but not the T-182C was a susceptibility factor in the brain morphology of MDD patients. Several case-control studies have investigated the potential association between the T-182C and MDD, but the results have been inconsistent and often contradictory. Inoue et al. demonstrated that there was a significant difference in the genotype distribution between MDD patients and HS in a Japanese population, and the C/C genotype was associated with lesser susceptibility to MDD [41]. However, a previous meta-analysis found no association between the T-182C polymorphism and MDD [42], which may support our negative result for the T-182C genotype. Furthermore, at the molecular biological level, the functional consequences of the T-182C remain obscure [9]. On the other hand, the G1287A has been associated with the CSF concentration of 3-methoxy-4-hydroxyphenylglycol (MHPG), a major norepinephrine metabolite [8]. They found that CSF MHPG concentrations were higher in HS with the G/G genotype than with A/- genotypes. Higher concentrations of MHPG may be attributable to a more active re-uptake of norepinephrine, resulting in lower norepinephrine levels in the G/G genotype participants than in the A/- genotype participants. Some studies have reported that the lower levels of norepinephrine impaired neuronal differentiation [43] because norepinephrine induces brain-derived neurotropic factor expression, which is the most prevalent growth factor in the central nervous system [44]. Therefore, we speculate that, in G/G genotype participants, the lower norepinephrine levels due to the active re-uptake of norepinephrine was related to the volume reductions in the PFC. Furthermore, the previous case-control study suggests that the G/G genotype of the G1287A may be involved in the development of MDD. Their analysis of the gene—environment interaction between the G1287A and their residency showed that Chinese rural women with the G/G genotype of the G1287A were susceptible to MDD [45]. These previous studies may support our findings in the MDD patients, suggesting that the integrity of the PFC in the G/G genotype participants might be more sensitive to the changes in brain norepinephrine than in the A/- genotype participants.

Some studies suggested that in MDD patients the G1287A may be useful for predicting their response to NET-targeted antidepressants. Yoshida et al. [46] found that the A/A genotype is associated with a lower response to serotonin-noradrenalin re-uptake inhibitors (SNRIs) than the G/A genotype because in patients with the A/A genotype the active re-uptake of norepinephrine is lower. Kim et al. [47] reported that patients with late-life depression who carry the G/G genotype showed better responses to norepinephrine re-uptake inhibitors (NRIs) compared with the A/- genotype participants, which suggests that NET plays an important role in pathologic conditions in the MDD patients with G/G genotype. Furthermore, our results indicate that the PFC abnormalities are present even in the early stage of MDD patients with the G/G genotype. Therefore, our observations suggest that the early intervention may be useful to prevent the brain changes during or before the first episode MDD with the G/G genotype.

Our study has some limitations. First, the number of participants was small. This made it impossible to explore potentially relevant interactions with other genotypes that affect the brain volume and it may have led to a positive bias. Longitudinal studies to explore the dynamics of the evolution of GM volume aberrations and to investigate their role in the disease prognosis and the response to treatment are underway. Second, in VBM analyses, we used the total GM volume as a covariate, but not a total intracranial volume (ICV), because we aimed to investigate the effect of *SLC6A2* on GM volume changes in MDD. The GM volume changes, such as hippocampus, caudate, prefrontal cortex, and posterior cingulate cortex, are well known to occur in MDD. Furthermore the automated-calculated ICV in SPM8 is less consistent with manual-calculated ICV compared to SPM12 and FreeSurfer [48, 49].

## Conclusion

In conclusion, the G1287A was associated with volume change of the PFC in patients experiencing the first episode and drug-naïve MDD patients. Thus, PFC aberrations may be at least partially related to the manifestation of MDD. However, it could be argued that the effect of one polymorphism of the gene fails to explain the morphological changes seen in MDD patients. We posit that in addition to the effects of the G1287A, other polymorphisms of MDD susceptibility genes and genotype—diagnosis interactions may affect the individual brain morphology. To elucidate relevant disease mechanisms we are in the process of exploring the effects of other neuromodulatory gene polymorphisms.
